# Discrimination Between Normal Skin Fibroblasts and Malignant Melanocytes Using Dielectrophoretic and Flow-Induced Shear Forces

**DOI:** 10.3390/mi16111232

**Published:** 2025-10-30

**Authors:** Yuta Ojima, Yuwa Takahashi, Shogo Miyata

**Affiliations:** 1 Graduate School of Science and Technology, Keio University, Yokohama 223-8522, Japan; ojima.epi.0930@gmail.com (Y.O.); yuuwa-takahashi@z6.keio.jp (Y.T.); 2 Faculty of Science and Technology, Keio University, Yokohama 223-8522, Japan

**Keywords:** dielectrophoresis, flow-induced shear force, cell diagnosis, skin cancer

## Abstract

Cell analysis is vital in clinical diagnostics and cell engineering research. Among the various analytical techniques, dielectrophoresis (DEP) is a particularly promising label-free method for distinguishing biological particles, which eliminates the need for fluorescent dyes or magnetic beads. In this study, we present a high-precision single-cell analysis system based on the evaluation of DEP forces in a controlled microfluidic flow environment. The system integrates a microfluidic chamber equipped with an electrode array to exert DEP forces and flow-induced shear forces to facilitate force balance analysis. We quantitatively characterized the DEP response to successfully discriminate between healthy skin cells and cancer cells using the proposed DEP-based cell-sorting platform. The proposed system successfully distinguished between these cell types even when their dielectrophoretic properties were similar, highlighting its potential for sensitive and selective cell classification in biomedical applications.

## 1. Introduction

The ability to accurately discriminate and separate cells is crucial in the biomedical and bioengineering fields, including clinical diagnostics, regenerative medicine, and drug discovery. In clinical settings, techniques such as staining-based cytology and polymerase chain reaction (PCR) are widely used to evaluate patient-derived tissue or fluid samples [1,2]. However, these methods typically require cell labeling, multistep preparation, and specialized expertise, which limit their applicability in rapid and label-free screening. Furthermore, cell labeling may affect cell viability and functionality, particularly in downstream applications.

Advanced cell separation techniques such as fluorescence-activated cell sorting (FACS) and magnetic-activated cell sorting (MACS) offer high throughput and specificity. FACS relies on fluorescent labeling and optical detection, while MACS uses magnetic beads conjugated with antibodies against cell surface markers [3,4,5]. Although both techniques are powerful, they require additional reagents and costly equipment, and have the risk of imposing mechanical or biochemical stress on cells. These limitations motivated the development of label-free and low-stress alternative methods.

Dielectrophoresis (DEP) has attracted considerable attention as a non-invasive, label-free method for manipulating and characterizing single cells based on their dielectric properties [6,7]. When exposed to nonuniform electric fields, polarizable particles, such as cells, experience DEP forces that depend on their morphology, membrane properties, and the conductivity and permittivity of the surrounding medium. The direction of the DEP force, either positive or negative, can be controlled by adjusting the frequency of the applied alternating current (AC) voltage, with the crossover frequency marking the point at which the force reverses its direction. The crossover frequency is determined by the Clausius–Mossotti factor, which depends on the electrical characteristics of the cell [8].

DEP has been used to distinguish different cell types, including healthy and cancerous cells, based on differences in their crossover frequencies [9,10,11,12,13,14,15]. However, when two cell types exhibit similar dielectric properties, such as in certain subtypes of healthy and malignant skin cells, conventional DEP analysis alone may fail to achieve sufficient discrimination. To address this problem, an auxiliary force, such as flow-induced shear force, can be introduced to enhance the separation resolution. By balancing the DEP force against the shear force in a microfluidic environment, the differences in response strength can be quantitatively evaluated, even when crossover frequencies are nearly identical.

In the present study, we developed and characterized a microfluidic cell analysis system capable of discriminating between normal skin fibroblasts and malignant melanocytes. The system integrates interdigitated electrodes for DEP force generation, and a controlled flow field to introduce shear forces. We demonstrated that the combination of these forces enabled fine discrimination between the two cell types, even under conditions in which DEP alone was insufficient.

## 2. Theory

### 2.1. Dieletrophoresis

DEP refers to the movement of dielectric particles, such as living cells, in a nonuniform electric field owing to an induced dipole moment [8]. For a spherical particle suspended in a medium, the time-averaged DEP force acting on the particle is given by

(1) $$F_{DEP}=2\pi r^{3}\varepsilon_{0}\varepsilon_{m}Re\left[f_{CM}\left(\omega \right)\right]\nabla E_{rms}^{2}$$

where *r*, *ε*_0_, *ε*_m_, Re [*f*_CM_], and *E*_rms_ denote the particle radius, vacuum permittivity, relative permittivity of the suspension medium, real part of the Clausius–Mossotti (CM) factor, and root mean square of the electric-field magnitude, respectively.

The CM factor describes the dielectric contrast between the particle and medium, and is defined as

(2) $$f_{CM}\left(\omega \right)=\frac{\varepsilon_{p}^{*}-\varepsilon_{m}^{*}}{\varepsilon_{p}^{*}+2\varepsilon_{m}^{*}}$$

where *ε*_p_* and *ε*_m_* are the complex permittivities of the particle and medium, respectively. These are given by:

(3) $$\varepsilon^{*}=\varepsilon_{0}\varepsilon -\frac{j\sigma }{\omega }$$

where *ε* is the relative permittivity of particle or medium; *σ* is the conductivity of particle or medium; *ω* is the angular frequency of the applied AC field; and *j* is the imaginary unit. The value of Re [*f*_CM_] determines the direction of the DEP force, with a positive value indicating positive DEP (p-DEP), where the particle is attracted to regions of high electric field intensity. Similarly, a negative value corresponds to negative DEP (n-DEP), where the particle is repelled.

In the proposed system, the DEP force was generated using a microfluidic chamber with a parallel-plate configuration: a top electrode formed from an Indium-Tin Oxide (ITO)-coated glass slide and a bottom electrode with slit-shaped Fluorine-doped Tin Oxide (FTO) electrodes patterned by laser etching. The height of a silicon rubber spacer defined the height of the chamber. The effective DEP force acting on a cell varies with its size and electrical properties, which are critical for discriminating between cell types with similar crossover frequencies.

The electric field distribution and the corresponding DEP force were numerically simulated using COMSOL Multiphysics Ver. 4.2a (COMSOL Inc., Stockholm, Sweden). The model assumed a two-dimensional cross-sectional geometry of the slit-patterned electrode (Figure S1). In the simulation, the cross section in the *xy*-plane was taken at the *z*-position that corresponds to the center of the microchannel. The *x*-direction spanned 100 μm between the centers of adjacent electrodes, utilizing the symmetry along the *x*-axis. The y-direction covered the entire chamber height of 500 μm. Here, *x* = 0 and *y* = 0 corresponded to the center of the electrode and height of the non-electrode surface, respectively. The DEP force vectors were calculated using the aforementioned formula, and cell size-dependent DEP force distributions were evaluated for radii of 5.5–11.5 μm. This analysis confirmed that the magnitude of the DEP force increased with the cell diameter (Figures S2–S4).

### 2.2. Flow-Induced Shear Force

In addition to the DEP force, fluid-induced shear stress is important for modulating cell motion within the chamber. When a laminar flow is generated across the slit electrodes using a syringe pump, wall shear stress *τ*_wall_ is exerted on the bottom surface and, consequently, on the attached or nearby cells. The wall shear stress in a rectangular channel can be approximated by

(4) $$\tau_{wall}=\frac{6\mu Q}{bh^{2}}$$

where *μ* is the dynamic viscosity of the buffer solution; *Q* is the volumetric flow rate; and *b* and *h* are the width and height of the flow channel, respectively.

**Figure 1:**
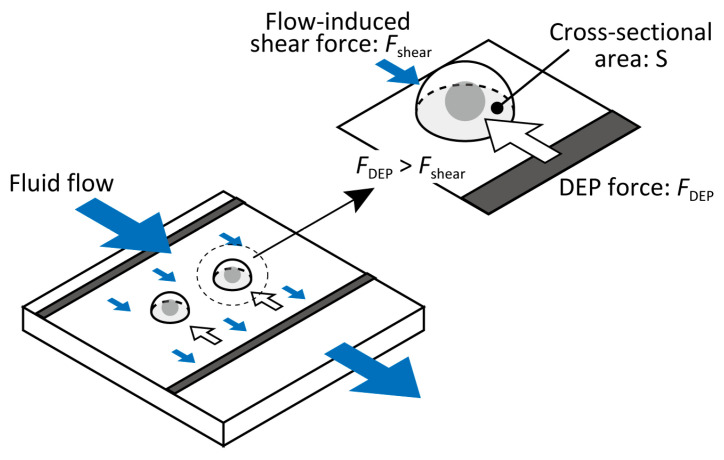
Schematic of the shear force acting on a hemispherical cell of diameter *d*, determined by integrating shear stress over contact area *S*.

Because most cells settle near the bottom owing to gravity and DEP attraction, fluid drag predominantly acts in the direction of the flow. The shear force *F*_shear_ acting on a hemispherical cell of radius *r* is approximated as the integral of the horizontal component of shear stress over the cell cross-sectional area (S)(, as illustrated in Figure 1.

The shear force (Fshear) acting on a hemispherical cell of radius r is approximated as the integral of the horizontal component of the shear stress over the cell’s cross-sectional area (S). This corresponds to the product of the wall shear stress and the cell’s cross-sectional area, as illustrated in Figure 1:

(5) $$F_{shear}=\pi r^{2}\tau_{wall}$$

The interplay between DEP and the flow-induced shear force enables the discrimination of cells not only by the direction of their DEP behavior (i.e., positive or negative), but also by the differences in the magnitude of DEP forces, and their force-dependent responses. In particular, when two cell types exhibit similar crossover frequencies and DEP polarities, the variation in how readily they respond to controlled shear stress can be leveraged to distinguish them with high precision.

## 3. Materials and Methods

### 3.1. Cell Culture

Human skin fibroblasts (SF-TY; Health Science Research Bank, Osaka, Japan) and human malignant melanocytes (G-361; Health Science Research Bank) were used in this study. Both cell lines were provided for research purposes under the approval and ethical oversight of the institutional ethics committee. They were cultured in Eagle’s Minimum Essential Medium (EMEM; Nacalai Tesque, Kyoto, Japan) supplemented with 10% fetal bovine serum (FBS; Gibco, Waltham, MA, USA) and a 1% antimycotic–antibiotic solution. The cultures were maintained in a humidified incubator at 37 °C and 5% CO_2_. Before the experiments, the cells were passaged 2–4 times after thawing the cryopreserved stock.

Before each experiment, cells were harvested with 0.25% trypsin–EDTA (Gibco) and resuspended in a low-conductivity (LC) buffer containing 10 mM HEPES, 0.1 mM CaCl_2_, 59 mM D-glucose, and 236 mM sucrose (conductivity: approximately 10 mS/m). The stability of the LC buffer, particularly the consistency of its electrical properties such as conductivity, has been verified in previous studies [13,14]. Furthermore, in our previous work [15,16,17,18,19], we confirmed that cell viability and function were maintained under the dielectrophoretic conditions employed in this study. The cell suspension was adjusted to a concentration of approximately 1 × 10^5^ cells/mL and used within 2 h of preparation.

### 3.2. DEP Chamber Design and Electric Field Simulation

**Figure 2:**
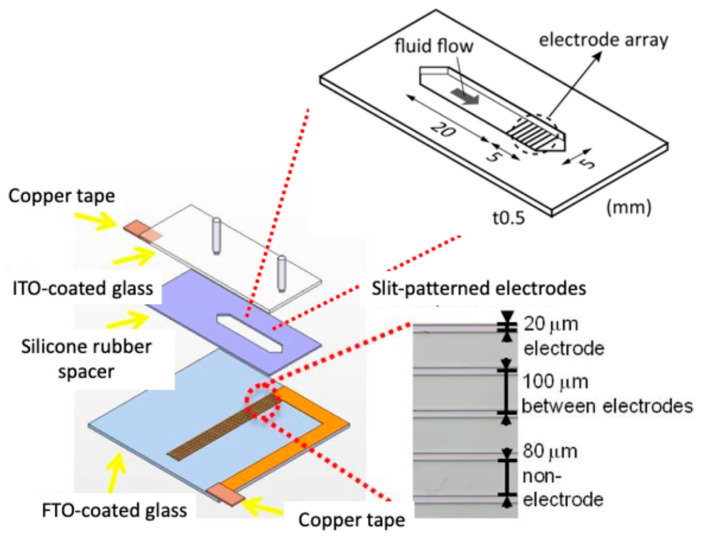
Schematic of the DEP chamber for the simultaneous application of DEP and flow-induced shear forces.

The DEP chamber was fabricated using two glass slides: an ITO-coated glass slide (Merck, Darmstadt, Germany) as the top electrode and an FTO-coated glass slide (Asahi Glass Co., Tokyo, Japan) as the bottom electrode. A silicone rubber spacer (thickness: 0.5 mm; width: 5 mm; channel length: 20 mm) was placed between them to form a rectangular flow chamber. On the FTO substrate, slit-type electrodes (width: 20 µm; gap: 80 µm; pitch: 100 µm) were patterned using a laser marker to generate nonuniform electric fields (Figure 2) [15].

### 3.3. Evaluation of DEP Response

**Figure 3:**
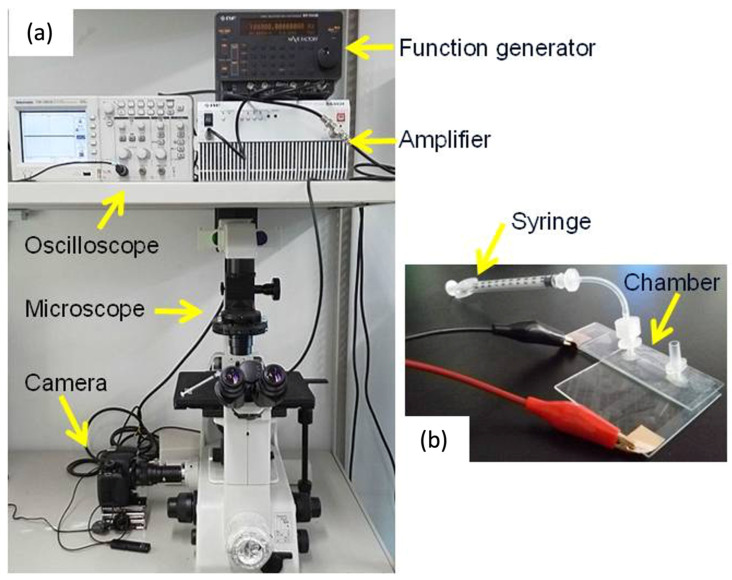
Experimental set-up for dielectrophoresis experiment (**a**) and DEP chamber (**b**).

An AC voltage (sinusoidal, 10 V_p–p_) was applied to the chamber using a function generator (WF1974, NF Corporation, Yokohama, Japan) and bipolar amplifier (BA4850, NF Corporation) (Figure 3). To evaluate the frequency-dependent DEP behavior of each cell type, the frequency was swept from 100 Hz to 1 MHz. Cell motion was observed using an inverted phase-contrast microscope (CKX41; Olympus, Tokyo, Japan) equipped with a digital CCD camera (DP70; Olympus, Japan). Prior to the dielectrophoresis experiments, the chamber was sterilized with 70% ethanol followed by rinses with 1% Pluronic F108 (Sigma-Aldrich, St. Louis, MO, USA). Pluronic 108 was used to avoid cell adhesion to the surface of glass slides and electrodes. Observations were performed over a 10 min period, and cell trajectories were recorded at 10 frames per second.

The numbers of cells on the electrodes (positive DEP) and between the electrodes (negative DEP) were counted. The frequency dependence of the DEP was evaluated by calculating the positive DEP (*R*_p_) and negative DEP (*R*_n_) ratios as follows:

(6) $$R_{p}=\frac{N_{p}}{N_{p}+N_{n}},\;R_{n}=\frac{N_{n}}{N_{p}+N_{n}}$$

where *N*_p_ and *N*_n_ denote the number of cells with positive and negative DEP behaviors, respectively. The crossover frequencies were determined by analyzing the change in direction of cell migration near the electrodes along with the frequency at which *R*_p_ and *R*_n_ intersected [15].

### 3.4. Application and Evaluation of Shear Force

A laminar flow was introduced through the DEP chamber using a syringe pump (PUMP33, Harvard Apparatus, Holliston, MA, USA). The flow rate was varied from 1 μL/min to 10 μL/min to modulate the wall shear stress from 0.12 × 10^−3^ Pa to 1.2 × 10^−2^ Pa, in increments of approximately 0.61 × 10^−3^ Pa. The flow was directed perpendicular to the slit electrode. The wall shear stress *τ*_wall_ was calculated using Equation (4), with *μ* = 1.519 × 10^−3^ Pa·s at 25 °C and a *Q* of 1–10 μL/min, while *b* and *h* were 5 mm and 0.5 mm, respectively. The shear force acting on individual cells was estimated using Equation (5).

The force was balanced by controlling the applied frequency and flow rate, and the critical shear stress at which the cells were displaced was used to infer the relative strength of the horizontal DEP force (Video S1). In this study, the applied frequencies used to evaluate the n-DEP forces were 500 Hz, 1 kHz, and 5 kHz.

### 3.5. Quantification of DEP Force

To estimate the absolute magnitude of the DEP force acting on the cells, force balance analysis was performed under static and dynamic flow conditions. Assuming that the cells were in equilibrium when the shear and horizontal DEP forces were counterbalanced, the magnitude of the DEP force was calculated as follows:

(7) $$F_{DEP}=F_{shear}$$

There is a possibility that this equilibrium assumption is affected by cell adhesive forces. As described above, the dielectrophoretic chamber was coated with Pluronic 108. This estimation was repeated at multiple frequencies for both cell types to evaluate the differential DEP responses.

### 3.6. Stastical Analysis

The data from the DEP response experiments are representative of three individual experiments using different cell culture populations. For each experimental group, 3 samples (*n* = 3) were analyzed, with each data point representing the mean and standard deviation.

For data of DEP force measurement, the statistical significance between SF-TY and G-361 cells was evaluated using the Tukey–Kramer test. The statistical significance was set at *p* < 0.05. The classification accuracy was evaluated based on the Mahalanobis generalized distance, which quantifies the separability between the two cell groups.

## 4. Results

### 4.1. Frequency-Dependent DEP Response and Crossover Frequencies

**Figure 4:**
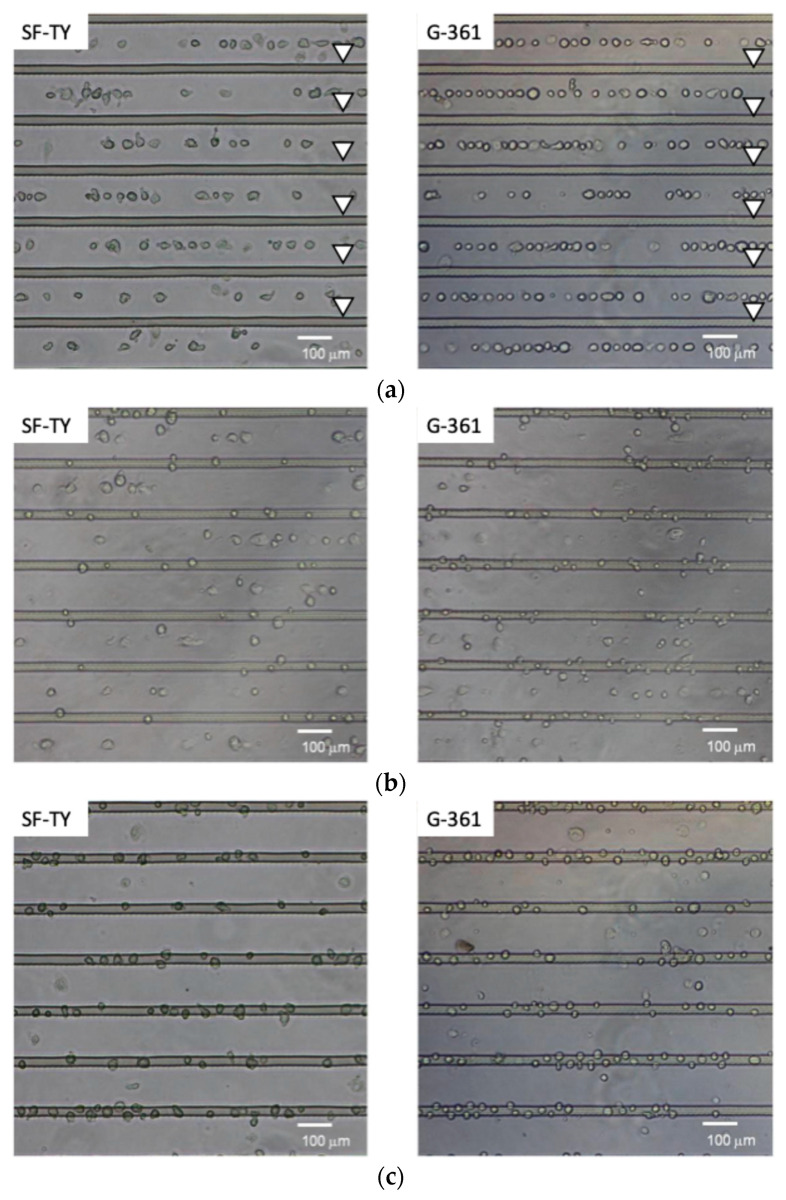
Microscopic images showing dielectrophoretic behavior at (**a**) 1 kHz, (**b**) 30 kHz, and (**c**) 100 kHz for SF-TY (**left**) and G-361 (**right**) cells. The arrowheads indicate the positions of the slit-patterned electrodes.

The DEP responses of SF-TY and G-361 cells were evaluated under sinusoidal AC electric fields across the frequency range of 1–100 kHz. At low frequencies (1 kHz and 5 kHz), both cell types predominantly exhibited negative DEP (n-DEP) behavior, with less than 10% of the cells accumulating at the electrodes (Figure 4a). At 100 kHz, more than 90% of both SF-TY and G-361 cells demonstrated positive DEP (p-DEP) behavior (Figure 4c). In the intermediate-frequency range (20–40 kHz), mixed responses were observed for both cell types, indicating the presence of crossover frequencies within this range (Figure 4b).

**Figure 5:**
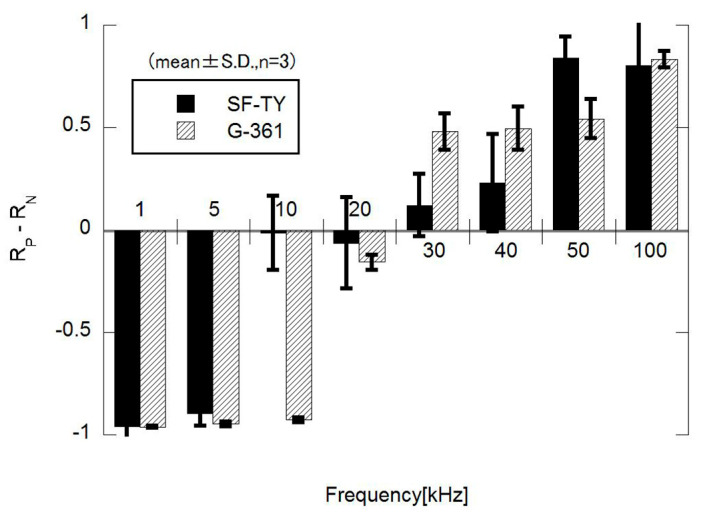
Frequency dependence of dielectrophoretic behavior, expressed as the difference between the proportions of cells exhibiting positive DEP (*R*_p_) and negative DEP (*R*_n_) behaviors. The data represent mean ± S.D. (*n* = 3).

The proportion of cells displaying p-DEP or n-DEP at each frequency was quantified from the microscopic images and summarized in the corresponding plots (Figure 5). These results indicated that the crossover frequencies of SF-TY and G-361 differed slightly and ranged between 20 kHz and 40 kHz. These slight differences limit the ability to discriminate between the two cell types based on the DEP polarity alone.

### 4.2. Evaluation of Cell Behavior Under Shear Flow

**Figure 6:**
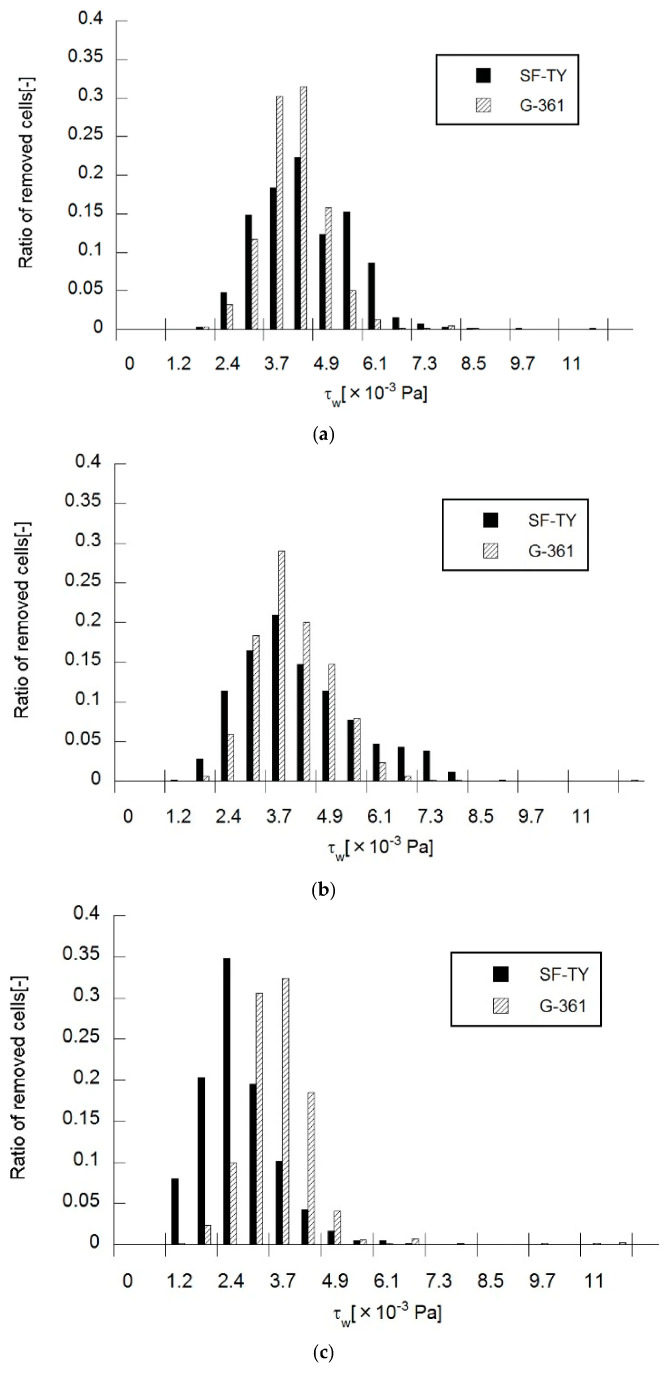
Ratio of cells removed by flow-induced shear stress under DEP at (**a**) 500 Hz, (**b**) 1 kHz, and (**c**) 5 kHz as a function of wall shear stress.

Shear stress-dependent cell removal was evaluated in SF-TY and G-361 cells at applied frequencies of 500 Hz, 1 kHz, and 5 kHz (Figure 6). At 500 Hz, SF-TY cells exhibited the highest removal rate (22%) at a shear stress of 4.3 × 10^−3^ Pa, while G-361 cells showed the highest removal rate (31%) at the same shear stress (Figure 6a). The classification accuracy under these conditions, determined from the Mahalanobis distance, was 55%, as shown in Table 1. At 1 kHz, the maximum removal rates for SF-TY cells (21%) and G-361 cells (30%) occurred at 3.7 × 10^−3^ Pa, with a classification accuracy of 53% (Figure 6b). At 5 kHz, SF-TY cells were most frequently removed (35%) at 2.4 × 10^−3^ Pa, whereas G-361 cells reached their maximum removal rate (32%) at 3.7 × 10^−3^ Pa (Figure 6c). The classification accuracy reached its highest value of 69% at this frequency.

Analysis of the mean removable shear stress (Figure 6, Table 1) revealed statistically significant differences between SF-TY and G-361 at the 0.1% level at 500 Hz and 5 kHz, and at the 2% level at 1 kHz. Both the cell types exhibited similar mean removable shear stresses at 500 Hz and 1 kHz. The difference in mean removable shear stress between the two cell types reached its maximum (0.9 × 10^−3^ Pa) at 5 kHz. This discrepancy in shear resistance suggests that despite exhibiting similar DEP polarities (e.g., negative DEP), the two cell types experience different magnitudes of the DEP force, which may be attributed to differences in the membrane structure or dielectric composition.

## 5. Discussions

This study demonstrated that combining dielectrophoretic and flow-induced shear forces enables the discrimination between SF-TY and (G-361), even when their frequency-dependent DEP responses appear qualitatively similar. While both cell types exhibited n-DEP behavior across much of the tested frequency range, a detailed analysis revealed differences in the crossover characteristics and response to shear stress.

The experiments for the DEP response evaluation revealed that the crossover frequency of SF-TY cells was slightly lower than that of G-361 cells in the frequency range of 20–40 kHz in which transitions between n-DEP and p-DEP occurred (Figure 5). For example, at 10 kHz, SF-TY cells exhibited a combination of p-DEP and n-DEP responses, whereas G-361 cells primarily exhibited n-DEP responses. This shift in the crossover frequency is likely influenced by differences in the cell size and membrane structure. According to DEP theory, a larger cell radius tends to reduce the crossover frequency because it affects the Clausius–Mossotti factor. Moreover, cell surface properties may also contribute to the observed frequency shift [8]. Similar to other cancer cells, malignant melanocytes (G-361) often express glycan-rich surface layers that are absent in normal fibroblasts [20,21]. These glycocalyx structures can increase the thickness of cell membrane to reduce the effective membrane capacitance, thereby increasing the crossover frequency relative to cells with a higher specific capacitance, such as SF-TY. To validate the hypothesis regarding the role of glycocalyx structures, detailed investigations of cell membrane architecture are required. In future studies, the micro- and nano-scale morphology of cell membranes should be examined using scanning electron microscopy (SEM) and transmission electron microscopy (TEM), and the presence of glycocalyx components should be evaluated through immunohistochemical staining with specific biomarkers. In this study, the theoretical framework of dielectrophoresis was applied without explicitly considering interfacial polarization with the electrochemical ion relaxation that occurs near the cell–medium interface. However, recent studies have reported that these interfacial effects, as described by the coupled theory of Maxwell–Wagner interfacial polarization and electrochemical ion relaxation, can play a significant role in determining the DEP response of biological cells [22,23,24]. Incorporating this theoretical framework in future analyses may also provide a more comprehensive understanding of the phenomena observed in this study.

Despite this frequency-based distinction, the crossover frequencies of the two cell types differed only slightly, which resulted in overlapping behavior across all frequency ranges and limited the ability to achieve a clear separation using DEP alone. To address this limitation, we evaluated the response to fluid shear stress under controlled DEP conditions as described in Section 4.2. Notably, under conditions such as a 5 kHz AC voltage and varying shear flow, SF-TY and G-361 cells showed markedly different detachment thresholds. This difference in susceptibility to displacement indicated that these cells showed different DEP forces even under the same AC frequency. The difference in amplitude of the DEP force could be related to the difference in the membrane structure between the healthy and cancer skin cells as mentioned above. High-resolution discrimination between the two cell types was enabled based on the differences in DEP forces. Conventional cell-sorting methods such as fluorescence-activated cell sorting (FACS) and magnetic-activated cell sorting (MACS) exhibit superior discrimination performance, achieving sorting accuracies of approximately 90% and 80%, respectively [25,26,27]. However, these techniques require labeling the cells with fluorescent dyes or antibodies, which may affect cell viability and limit their applicability in clinical settings. In contrast, the DEP force–based approach proposed in this study enables label-free discrimination of cells. Although its discrimination accuracy has not yet reached the level required for practical clinical application, the ability to distinguish cell types without labeling is a notable advantage of this method. Furthermore, by introducing a dimensionless evaluation parameter defined from the fluid-induced shear force, dielectrophoretic force, and cell diameter, the generality and applicability of this approach could be further enhanced.

Our results highlight that dual-modality evaluation using both dielectric- and mechanical-force-based criteria provides a more robust approach for cell classification than DEP alone. Furthermore, the discrimination accuracy of this method could be enhanced by optimizing the electrode pitch and integrating impedance measurements, an approach that has already been demonstrated by previous studies [23,24,28], thereby extending its applicability to the analysis of subtle cellular differences in label-free biomedical diagnostics and cell engineering applications.

## 6. Conclusions

In this study, we developed and evaluated a microfluidic system combining DEP and flow-induced shear forces to discriminate between SF-TY and G-361. Frequency-dependent analysis revealed that both cell types exhibited negative DEP behavior at low frequencies. However, subtle differences in their crossover characteristics were observed, with SF-TY cells showing a tendency toward lower crossover frequencies.

To enhance the discrimination, we introduced a controlled shear flow and quantified the critical shear stress required to detach the cells from the electrode region under fixed DEP conditions. This force-based evaluation revealed a consistent difference in the detachment thresholds between the two cell types, allowing high-resolution classification even in cases with similar dielectric responses.

These findings demonstrate that integrating DEP with hydrodynamic force analysis provides a powerful label-free approach for distinguishing between phenotypically similar cells. While the sorting accuracy of this approach remains lower than that of established techniques such as FACS and MACS, it nonetheless demonstrates promising potential for broader applications in single-cell diagnostics, cancer screening, and biophysical phenotyping using microfluidic platforms.

## Figures and Tables

**Table 1:** Removal responses and predictive discrimination ratios between SF-TY and G-361 cells.

Frequency of AC Electric Field (kHz)	Cell Line	Number of Samples	Removal Shear Stress (×10^−3^ Pa)	Classification Accuracy (%)	Significant Difference
0.5	SF-TY	566	4.4 ± 1.2	55	*p* < 0.001
	G-361	616	4.1 ± 0.8		
1	SF-TY	596	4.2 ± 1.4	53	*p* < 0.02
	G-361	631	4.0 ± 1.0		
5	SF-TY	614	2.6 ± 0.9	69	*p* < 0.001
	G-361	713	3.5 ± 0.9		

## Data Availability

The data presented in this study are available on reasonable request from the corresponding author.

## Supplementary Materials

The following supporting information can be downloaded at: https://www.mdpi.com/article/10.3390/mi16111232/s1. Figures S1–S4: Numerical analysis of electric field in the dielectrophoretic chamber. Video S1: Cell movement under negative dielectrophoresis and flow-induced shear forces.
