# Supplementary material for: Discrimination Between Normal Skin Fibroblasts and Malignant Melanocytes Using Dielectrophoretic and Flow-Induced Shear Forces
Source: Micromachines (Basel). 2025 Oct 30;16(11):1232. doi: 10.3390/mi16111232 (PMC12654393; doi:10.3390/mi16111232)
Supplement: Supplementary file 1 [file micromachines-16-01232-s001.zip › micromachines-3876791-supplementary/micromachines-Ojima tumor analysis_DEP_Suppl_figs.pdf]

Discrimination between Normal Skin Fibroblasts and Malignant Melanocytes Using Dielectrophoretic and Fluid-Induced Shear Forces.

Yuta Ojima [1], Yuwa Takahashi [1], and Shogo Miyata [2],*

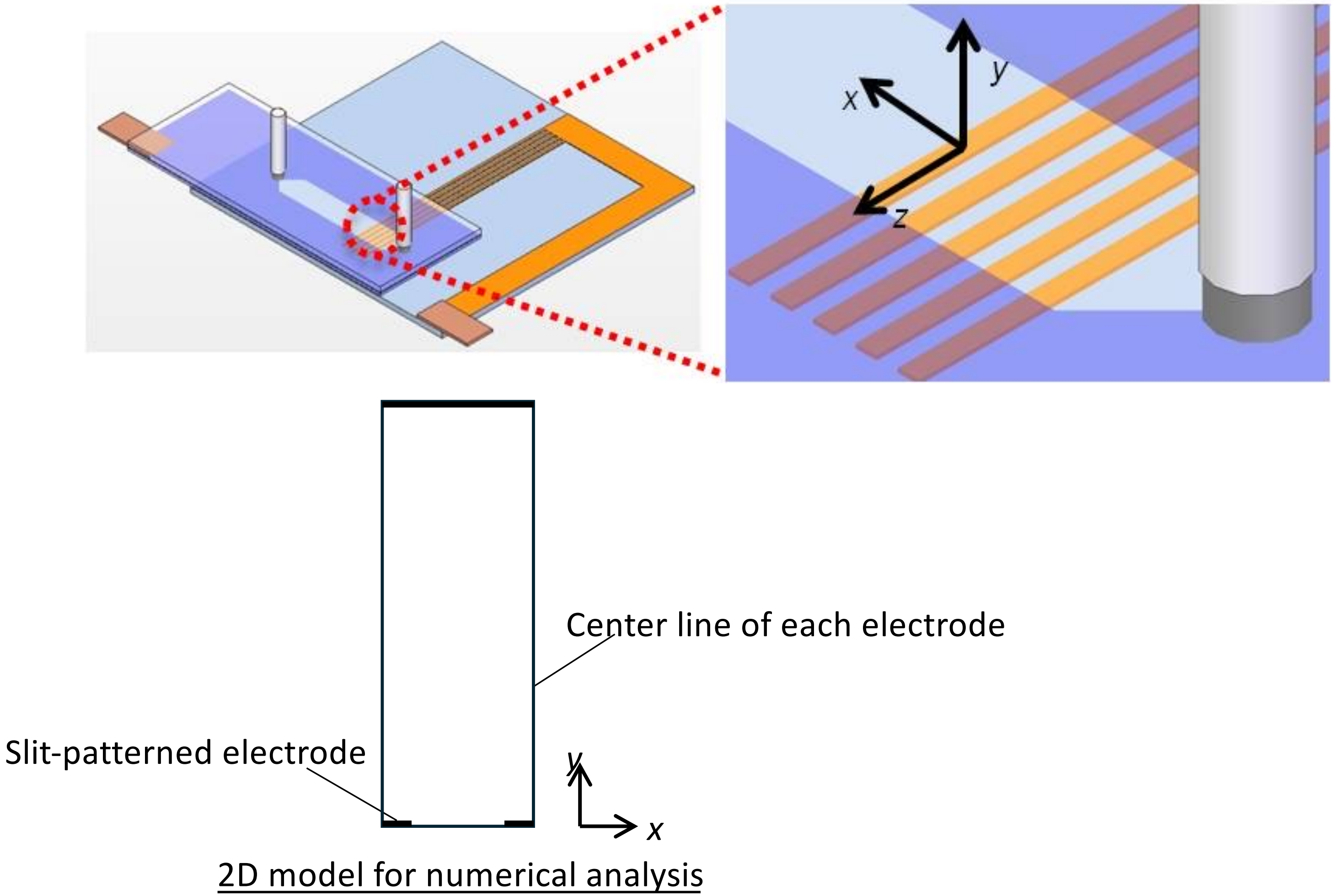

Supplemental: Figure S1, Cross-sectional numerical model of dielectrophoresis chamber.

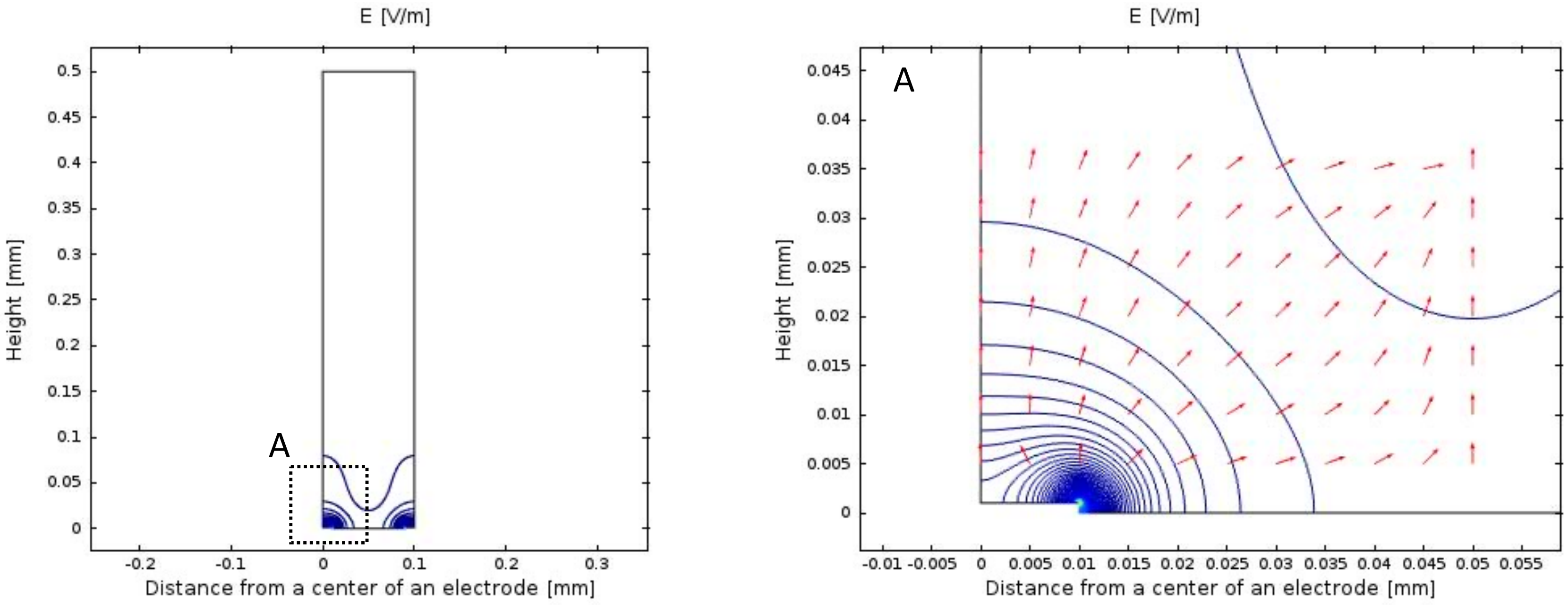

Supplemental: Figure S2, Electric field distribution in the dielectrophoresis chamber (left) and magnified view near the electrodes (A) showing the electric field distribution and directions of negative DEP forces (right).

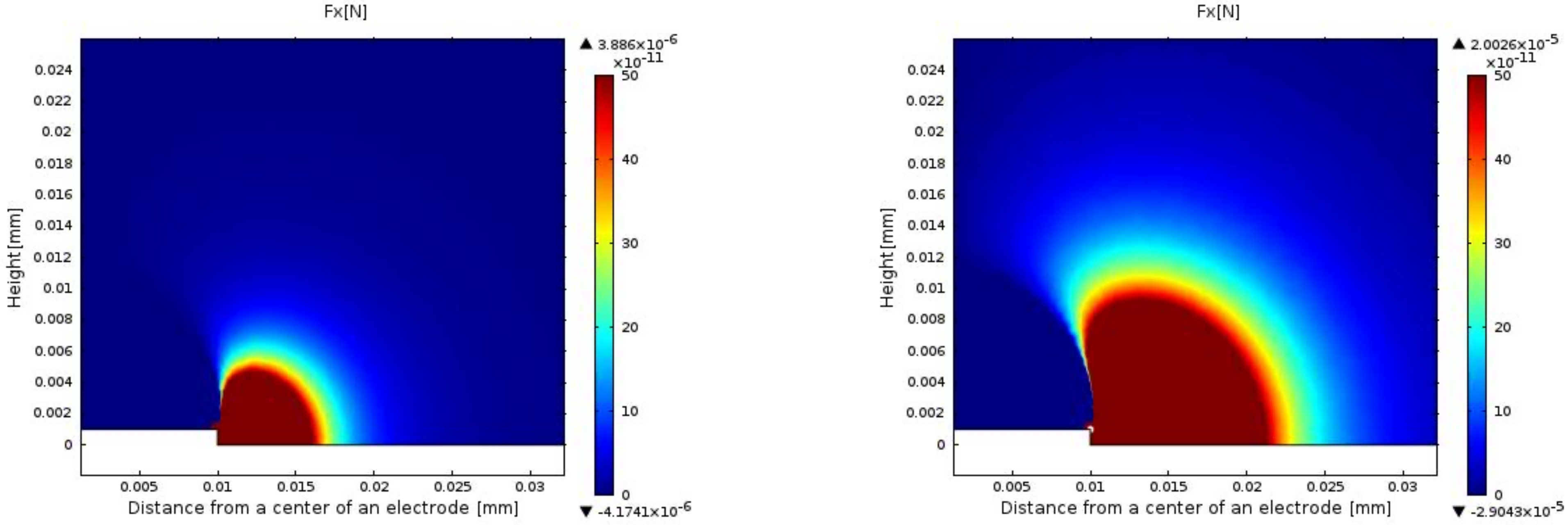

Supplemental: Figure S3, Distribution of dielectrophoretic (DEP) force magnitude around the electrode surface in the DEP chamber. (A) Cell radius = 5.5 µm; (B) Cell radius = 11.5 µm.

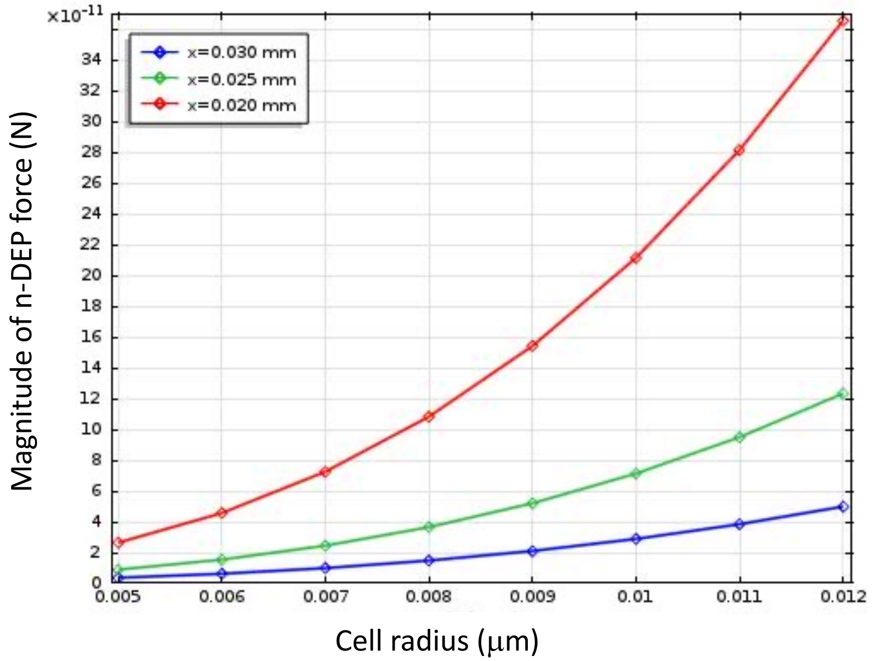

Supplemental: Figure S4, Effect of cell radius on magnitude of negative dielectrophoretic (DEP) force, as obtained from numerical simulation.
