# Supplementary material for: Discrimination Between Normal Skin Fibroblasts and Malignant Melanocytes Using Dielectrophoretic and Flow-Induced Shear Forces
Source: Micromachines (Basel). 2025 Oct 30;16(11):1232. doi: 10.3390/mi16111232 (PMC12654393; doi:10.3390/mi16111232)
Supplement: Supplementary file 1 [file micromachines-16-01232-s001.zip › micromachines-3876791-supplementary/micromachines-Ojima_tumor_analysis_DEP_Suppl_subm2.pdf]

Supplementary

S.1 Numerical analysis of electrical field and DEP force generated in dielectrophoretic chamber

In this study, numerical simulations were performed using COMSOL Multiphysics Version 4.2a (COMSOL Inc., Sweden). The parameters used for the dielectrophoretic (DEP) chamber analysis are listed in Table S1, and the coordinate system was defined as shown in Fig. S1.

To evaluate the DEP force generated near the electrodes of the chamber, a cross-sectional plane was taken at the center of the flow channel in the z-direction, and the x- and y-components of the DEP force were analyzed numerically. Taking advantage of symmetry in the x-direction, the analysis region was set to 100 μm from the center of one electrode to the center of the adjacent electrode, and 500 μm in the y-direction corresponding to the channel height. The electric field intensity and DEP force distributions around the electrodes were evaluated within this domain. In the simulation results, $x = 0$ corresponds to the electrode center, and y = 0 represents the height of the non-electrode surface. The DEP force imposed on cell was calculated using formula (1) and the results of electrical field analysis. Because the cells were suspended in the medium during dielectrophoresis, the analysis was conducted under the assumption of a spherical cell model.

Table S1. Parameters used for the electrical fields and DEP force analyses in the dielectrophoretic (DEP) chamber analysis.

Parameters	Number of samples
Medium viscosity μ (Pa·S)	1.519×10^{-3}
Medium conductivity σ (S/m)	9.89×10^{-3}
Medium relative permittivity ε_m (-)	80
Hight of DEP chamber h (mm)	0.5
Width of DEP chamber w (mm)	5
Width of electrode W (μm)	20
Hight of electrode H (μm)	0.05
Electrode pitch L (μm)	100
Applied voltage V^+ (V)	10
Applied voltage V^- (V)	-10
Clausius-Mossotti factor $\mathrm{Re}\left[\widehat{K}(\omega)\right]$ (-)	0.5

The results of the numerical simulation for the electric field distribution in the dielectrophoretic (DEP) chamber, the detailed field distribution near the electrodes, and the

direction of the negative DEP force are shown in Fig. S2. In Fig. S2 (left part), the rectangular region represents the cross section of the chamber in the *xy*-plane, where the electrodes are located at the lower left and lower right of the chamber bottom. The simulation results indicate the equipotential lines of the electric field. Figure S2 (right part) shows an enlarged view of the region near the lower-left electrode, with the electrode positioned at the lower left corner. The arrows in the simulation image represent the direction of the negative DEP force at each coordinate. The strong electric fields are scarcely present near the upper surface of the chamber, whereas the regions of high field intensity are concentrated near the electrodes at the bottom surface. The electric field is strongest around the upper-right edge of the electrode, where the equipotential lines are densely distributed in the y-direction. Figure S3 shows the distribution of the magnitude of *x*-component of the negative dielectrophoretic (DEP) force, $|\boldsymbol{F}_{DEP},x|$, in an enlarged view of the region near the lower-left electrode. It was found that the magnitude of the negative DEP force increases with increasing cell radius, *r* and decreased with increasing in the distance from the electrode, *x* (Fig. S4). However, it should be noted that this simulation was conducted under the assumption that cells are perfectly spherical. Therefore, the calculated DEP forces may not fully represent the actual forces acting on real cells, which often exhibit irregular shapes and surface features.

S.2 Movement of living cells under dielectrophoresis and fluid-induced shear force

In this study, the movement of living cells was recorded using a CMOS camera mounted on an inverted microscope.

Initially, the cells were repelled from the electrodes due to negative dielectrophoresis (n-DEP) and moved toward the region between the electrodes under a 5 kHz AC electric field. Subsequently, they were pushed toward the electrodes until a balance was achieved between the repelling n-DEP force and the fluid-induced shear force. When the shear force exceeded the n-DEP force, the cells were displaced. This critical shear force was regarded as the magnitude of n-DEP force imposed on the cell. As shown in Video S1, cells were displaced at different shear force values depending on their individual dielectrophoretic properties.
